# Low-density lipoprotein cholesterol and risk of COPD: a Mendelian randomization study

**DOI:** 10.1038/s41598-026-48823-6

**Published:** 2026-04-15

**Authors:** Josefine Freyberg Justesen, Benjamin Nilsson Wadström, Sarah Caroline Weisenfeldt Marott, Eskild Morten Landt, Børge Grønne Nordestgaard, Shoaib Afzal, Morten Dahl

**Affiliations:** 1 Department of Clinical Biochemistry, Zealand University Hospital, Lykkebaekvej 1, Køge, Denmark; 2 Department of Clinical Biochemistry, Copenhagen University Hospital - Herlev Gentofte, Herlev, Denmark; 3 The Copenhagen General Population Study, Copenhagen University Hospital - Herlev Gentofte, Herlev, Denmark; 4 Department of Clinical Medicine, University of Copenhagen, Copenhagen, Denmark

**Keywords:** COPD, COPD exacerbation, COPD-specific mortality, Spirometric COPD, LDL cholesterol, Mendelian randomization., Diseases, Genetics, Medical research, Risk factors

## Abstract

Statins have shown effects on exacerbation rates and lung function in individuals with chronic obstructive pulmonary disease (COPD) in randomized controlled trials. This has led to speculation that elevated LDL cholesterol may increase the risk of adverse COPD outcomes. We tested the hypothesis that high LDL cholesterol is causally associated with increased risk of severe COPD exacerbation, COPD-specific mortality, and spirometric COPD through a one-sample Mendelian randomization study design using individual-level data in two cohorts. We examined 108,438 adults from the Copenhagen General Population Study (CGPS). A weighted allele score was calculated using nine biologically relevant genetic variants associated with LDL cholesterol levels, and causal risk estimates for the three outcomes were determined using instrumental variable analysis. Findings were validated using one-sample Mendelian randomization in 389,627 individuals from the UK Biobank (UKB). Causal odds ratios from the CGPS were not statistically significant for severe COPD exacerbation (odds ratio (OR) 1.12 (95% CI: 0.88–1.42), *p* = 0.34) or COPD-specific mortality (OR 1.08 (0.69–1.69), *p* = 0.74). Similar findings were seen in the UKB. Causal estimates showed a decreased risk of cross-sectional spirometric COPD with 1 mmol/L (39 mg/dL) higher LDL cholesterol in the CGPS (OR 0.78 (0.66–0.92), *p* < 0.001); however, causal estimates were not statistically significant in the UKB (OR 1.03 (0.95–1.11), *p* = 0.50). Sensitivity analyses using MR Egger, inverse-variance weighted, weighted median, and weighted mode methods did not indicate a true causal association for any outcome in either cohort. In conclusion, in the CGPS and UKB, high LDL cholesterol did not causally increase the risk of severe COPD exacerbation and COPD-specific mortality. Further, our findings do not provide consistent evidence to support a causal role of high LDL cholesterol in reducing the risk of developing COPD.

**Supplementary Information:**

The online version contains supplementary material available at 10.1038/s41598-026-48823-6.

## Background

Chronic obstructive pulmonary disease (COPD) is a preventable and treatable respiratory disease, but is, however, the fourth leading cause of death with an estimated 10% prevalence worldwide^1–3^. Current medical treatments, such as beta-adrenergic and corticosteroid inhalations, do not sufficiently affect the progression of COPD, as individuals with COPD experience approximately one to four exacerbations annually^4^. Statins investigated in individuals with COPD have been shown to decrease rates of exacerbations and increase lung function^5,6^. Though the primary effect of statins is their low-density lipoprotein (LDL) cholesterol-lowering effect, the effect on COPD has been attributed to pleiotropic effects, such as anti-inflammatory and anti-oxidative properties, though this has not been clearly established^7–10^. As lowering of LDL cholesterol has shown a protective effect on COPD, it could be hypothesized that LDL cholesterol might have a causal effect on COPD, potentially through increased levels of oxidative stress and oxidized LDL cholesterol induced by smoking. Inflammation, oxidative stress, and decreased lung function have been associated with oxidized LDL cholesterol in individuals with COPD^11^. Our group has previously investigated the association between high levels of LDL cholesterol and COPD outcomes observationally. Surprisingly, we found that low levels of LDL cholesterol were associated with an increased risk of severe COPD exacerbation and COPD-specific mortality, though these associations were thought to be a result of reverse causation^12^.

Mendelian randomization (MR) is an epidemiological approach used to address causality and largely bypass confounding^13^. MR studies utilize the random distribution of alleles in homogenous populations and that an individual’s genotype always precedes any outcome of interest^14^. When comparing genetic variants associated with an exposure of interest, it is thereby possible to ascertain causality between the exposure and outcome of interest without interference from reverse causality and with minimal confounding.

In this study, we aimed to test the hypothesis that high LDL cholesterol is causally associated with severe COPD exacerbation, COPD-specific mortality, and spirometric COPD. We tested these hypotheses in the Copenhagen General Population Study (CGPS) using individual-level data on nine genetic variants in the biologically relevant genes *APOB*, *LDLR*, *PCSK9*, *HMGCR*, and *NPC1L1* in a one-sample MR study design and validated our findings in the UK Biobank using genetic variants in the same five genes identified by a previously published genome-wide association study (GWAS)^15^. As cardiovascular disease (for which LDL cholesterol is a critical treatable causal risk factor) is the most prevalent comorbidity in individuals with COPD^16,17^, knowledge of the effect of LDL cholesterol on COPD is of great clinical importance.

## Methods

### Study population

Data were ascertained from 108,438 individuals with data on LDL cholesterol (n = ≤ 3 excluded with missing data) enrolled in the CGPS, a population-based cohort study representing the Danish general population. The CGPS was initiated in 2003 and continues with ongoing enrolment. Participants were invited by random selection on the basis of the national Danish Civil Registration System to reflect the Danish population aged 20 to 100. All participants were of white European Danish descent.

### LDL cholesterol and covariates

On the day of enrolment, blood samples were collected in nonfasting state for lipid measurements in accordance with international guidelines^18,19^. Plasma LDL cholesterol was measured using standard hospital assays. LDL cholesterol was calculated using the Friedewald equation when plasma triglyceride concentration was ≤ 4 mmol/L (354 mg/dL) and measured directly using a direct enzymatic method (Thermo Fisher Scientific/Konelab) when plasma triglycerides were > 4 mmol/L (354 mg/dL)^20^. When participants received lipid-lowering medication, LDL cholesterol was multiplied by 1.43 to adjust for an approximately 30% decrease in LDL cholesterol concentration^12,21,22^. A comparison of exposure, genetic instrument, and outcomes used in the two cohorts, the CGPS and the UK Biobank, can be seen in Supplementary Table 1. Prebronchodilatory spirometry was performed using EasyOne Spirometer (NDD Medical Technologies) for measures of forced expiratory volume in the first second (FEV_1_) and forced vital capacity (FVC)^23^. Participants completed a self-reported questionnaire at baseline, including information on use of medication, history of disease, physical activity, alcohol consumption, and smoking habits. Physical inactivity was defined as light physical activity during leisure time for four hours or less per week. Body mass index (BMI) was calculated as measured weight in kilograms divided by measured height in meters squared. Height and weight were measured at baseline. Alcohol was defined as 1 item = 12 g of alcohol and dichotomized to > 10 items/week as is recommended by the Danish Health Authority.

### Genotypes and weighted allele score

Nine genetic variants previously associated with high levels of LDL cholesterol were genotyped at Department of Clinical Biochemistry, Herlev Gentofte Hospital, using an ABI PRISM 7900HT Sequence Detection System (Applied Biosystems Inc., Foster City, California) and Taqman-based assays: *APOB* R35000Q (rs5742904); *LDLR* W23X (rs267607213), W66G (rs121908025), and W556S (rs138947766); *PCSK9* R46L (rs11591147), V474I (rs562556), and E670G (rs505151); *HMGCR* (rs17238484); and *NPC1L1* (-18(rs41279633))^24,25^. To limit the risk of horizontal pleiotropy, variants were specifically chosen in genes with known roles in the LDL metabolism. For illustrative purposes, the weighted allele score was divided into five groups to indicate a stepwise increase in LDL cholesterol level used for estimates of genetically observed risk of COPD outcomes. A weighted allele score was calculated for each participant as the sum of weights of LDL cholesterol increase per allele, see Supplementary Table 2. Validity of the genetic instrument was evaluated by an F-statistics value of 1,717, as a value > 10 suggests sufficient strength^14,25^, and an R^2^ of 1.6%. An alternative weighted allele score used for sensitivity analyses were calculated to minimize bias from statin use and the risk of overfitting. Description of calculation of this alternative weighted allele score is seen in Supplementary Appendix 1.

### Outcomes

Spirometric COPD was defined as FEV_1_/FVC < 0.7 measured at baseline. Individuals stating that they had asthma in the questionnaires were excluded from the spirometric COPD variable.

Prospective outcomes (Severe COPD exacerbation, COPD-specific mortality, ischemic heart disease) were collected from the national Danish Patient Registry from January 1977 until December 2018 and from the national Danish Cause of Death Registry until December 2019; if participants died or emigrated during follow-up, they had their follow-up truncated in statistical analyses at the exact date of death or emigration (such information is 100% complete in the Danish health registries). As recommended by the Danish Register of COPD, severe COPD exacerbation was defined as a hospital admission with a registered primary diagnosis of International Classification of Diseases (ICD)10 J44, or ICD10 J44 as a secondary diagnosis with respiratory failure (ICD10 J96) or pneumonia (ICD10 J18) as a primary diagnosis^12,26^. COPD-specific mortality was defined as ICD10 J41-44 registered in the national Danish Cause of Death Registry as a primary cause of death. Ischemic heart disease used as a positive control was defined as having a hospital admission with a primary diagnosis of ischemic heart disease (ICD8: 410–414, ICD10: I20-I25).

### Validation in the UK biobank

The UK Biobank is an extensive British prospective study investigating genetic and environmental factors for major diseases^27^. 500,000 participants aged 40–69 years old were recruited, and baseline data were collected between April 2007 and ultimo 2010^27^. General information has been described previously^28^. Baseline data include questionnaires, physical and cognitive measurements, and samples of blood and urine. We included 389,627 individuals of white European Ancestry (UK Biobank Data-Field 22006, *n* = 389,627) with data on LDL cholesterol (LDL > 0 mmol/L, *n* = 468,403) measured directly by enzymatic protective selection analysis on a Beckman Coulter AU5800 (Data-Field 30780) accessed through application #104,807. Prebronchodilatory spirometry measurements were performed using the Vitalograph Pneumotrac 6800 spirometer (Vitalograph, Maids Moreton, UK), and spirometric COPD was defined as FEV_1_/FVC < 0.7 excluding participants with self-reported asthma, as in the CGPS (see Supplementary Table 1). Data on prospective outcomes were collected through British national registries, and outcomes were defined similarly to the CGPS (see Supplementary Table 1).

For the primary weighted allele score, SNPs that were (i) in the proximity (+/- 100 kb) of the *APOB*, *LDLR*, *PCSK9*, *HMGCR*, and *NPC1L1* genes; (ii) had R^2^ < 0.1; and (iii) were associated with LDL cholesterol at *p* < 5 × 10^− 6^ in a previous GWAS of European ancestry individuals aged < 50 years from the UK Biobank were included^15^. The *p* value and R^2^ thresholds were higher than the conventional genome-wide thresholds due to selection from a lower number of variants in close proximity, while only individuals aged < 50 years were included since these have a low prevalence of statin use to minimize bias from statin use and overfitting while avoiding collider bias, as weights were applied for individuals of all ages^29^. Potential bias introduced from this partial sample overlap is expected to be negligible. Adjustments were for age, age^2^, sex, sex*age, fasting time, and the first 40 principal components. The weights for LDL cholesterol change per allele from this GWAS were used to calculate a weighted allele score for each individual, see Supplementary Table 3 Further information on the GWAS and validation of the genetic score is previously described^15^. An alternative weighted allele score used for sensitivity analyses was calculated to gain a higher percentual contribution of the variation in LDL cholesterol. For information on calculation of this alternative weighted allele score, see Supplementary Appendix 2.

### Mendelian randomization and instrumental variable analysis assumptions

**Fig. 1:**
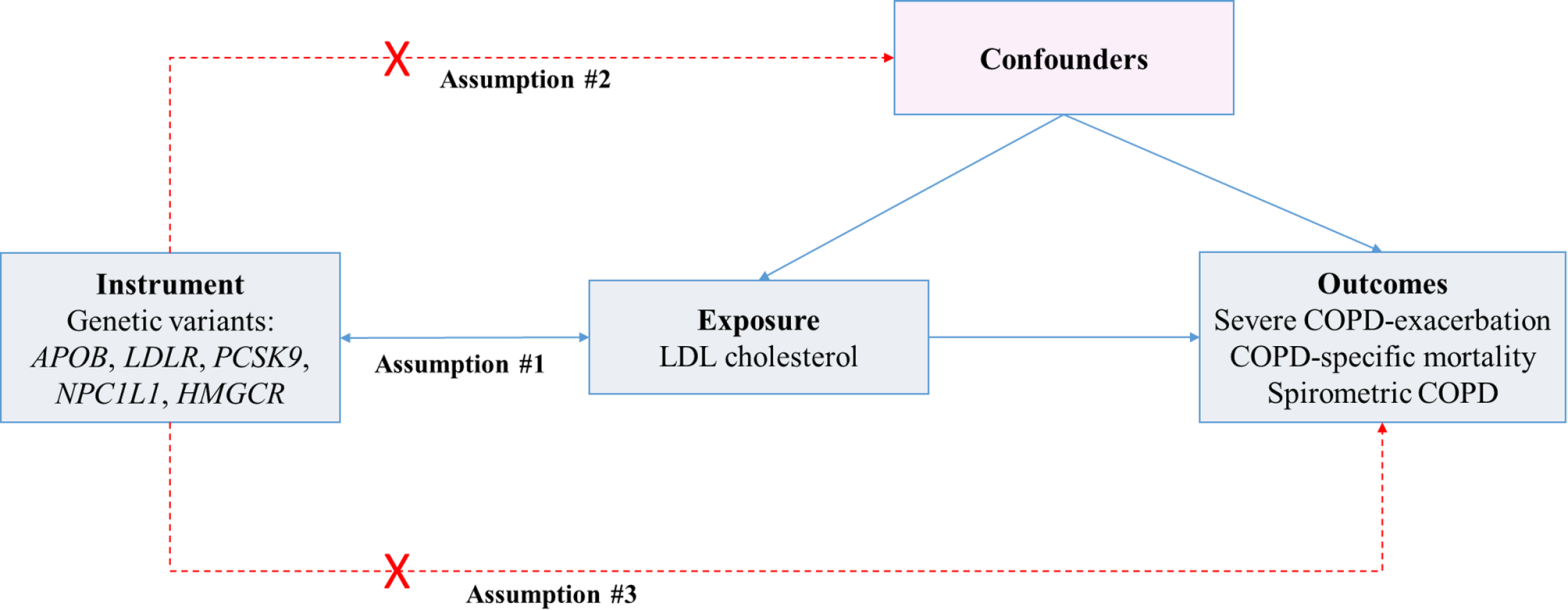
Diagram illustrating the three key assumptions of instrumental variable analysis. #1 The relevance assumption: The genetic variants of *APOB*, *LDLR*, *PCSK9*, *HMGCR*, and *NPC1L1* must be robustly associated with the exposure LDL cholesterol. #2 The independence assumption: Genetic variants are independent of known confounders of the exposure-outcome association. #3 The exclusion restriction assumption: The genetic variants included must have an effect on the outcomes only through the exposure.

The MR study design utilizes instrumental variable analysis to establish causality for lifelong and modifiable exposures, and as such does not reflect short-term intervention effects. For MR studies three key assumptions must be fulfilled^14^. Firstly, the genetic variants must be associated with the exposure of interest, meaning that the included variants of *APOB*, *LDLR*, *PCSK9*, *HMGCR*, and *NPC1L1* must be associated with LDL cholesterol concentration (The relevance assumption; Fig. 1, #1). Secondly, the genetic instruments must not be associated with known confounders (The independence assumption; Fig. 1, #2). Thirdly, the genetic instruments must have an effect on the outcomes only through the exposure, i.e., genetic variants do not exhibit horizontal pleiotropy (The exclusion restriction assumption; Fig. 1, #3). Supplementary Table 4 contains an overview of the assessment of each assumption.

### Statistics

Data were analysed using Stata/MP v. 16.0 and 18.0. Pearson’s χ^2^ test was used to evaluate Hardy-Weinberg equilibrium for all genotypes using “*hwsnp”* in Stata. Genotype data with missing values were excluded from analyses. Missing data on covariates were not imputed.

Firstly, to investigate the association of weighted allele scores with COPD outcomes, we used logistic regression, Cox proportional hazard regression with entry at birth and age as time-scale, and Fine and Gray sub-distribution hazard model with death as competing risk. All regressions were adjusted for age and sex. To investigate the independence assumption (Fig. 1, #2), associations of potential confounders and LDL cholesterol or weighted allele score were estimated using unadjusted logistic regression^25,30^. Estimates for associations of potential confounders were deemed statistically significant at a threshold of *p* < 0.006 (0.05/9) by Bonferroni correction for nine multiple comparisons.

Secondly, to ascertain causal risk estimates, unadjusted instrumental variable analysis using the generalized method of moments estimator through the *ivpoisson* command in Stata was used for 1 mmol/L higher LDL cholesterol with weighted allele score as a continuous variable. Causal risk estimates for cross-sectional spirometric COPD were calculated in individuals > 50 years of age to minimize the risk of overfitting data and bias of statin treatment. To validate findings in the UK Biobank, the *ivpoisson* command was likewise applied in Stata in the DXJupyterLab platform for causal estimates, although estimates were adjusted for the first 10 principal components (Data-Field 22009) and genotype chip (Data-Field 22000). MR estimates were deemed significant at a threshold of *p* < 0.017 (0.05/3) by Bonferroni correction for three comparisons. Sensitivity analyses were conducted (1) by use of alternative weighted allele scores to minimize bias or increase power and (2) as MR Egger, inverse-variance weighted (IVW) MR, weighted median, and weighted mode method using generated summary statistics for each genetic variant in both cohorts to address the exclusion restriction assumption (Fig. 1, #3). For the latter, MR estimates were deemed significant at a threshold of *p* < 0.0125 (0.05/4) by Bonferroni correction for the four sensitivity analyses (MR Egger, IVW, weighted median, and weighted mode method).

Thirdly, to calculate risk estimates (odds and hazard ratios) for COPD outcomes by observational LDL cholesterol, we used logistic regression and Cox proportional hazard regression with delayed entry (left truncation) at study examination and age as underlying time-scale. Regressions were multivariable adjusted for age, sex, BMI, alcohol consumption as a continuous variable, physical inactivity, smoking status, and pack-years.

## Results

In 108,438 individuals from the CGPS, there were 2,415 individuals with severe COPD exacerbation during a median of 69 years of follow-up (Table 1). The median age was 70 years (interquartile range [IR]: 63–77) versus 58 years (IR: 48–67) for those with versus without severe COPD exacerbation. Furthermore, smoking prevalence and amount were higher in the severe COPD exacerbation group, while lung function was lower. 13,098 individuals died (all-cause), and 899 emigrated before having an event or reaching the end of the study.

**Table 1:** Characteristics of 108 438 individuals from the CGPS by severe COPD exacerbation.

No. of individuals, %	Severe COPD exacerbation	No Severe COPD exacerbation
	2 415 (2.2)	106 023 (97.8)
Age, years	70 (63–77)	58 (48–67)
Women, %	1 294 (53.6)	58 328 (55.1)
LDL cholesterol, mmol/L	3.2 (2.7–3.9)	3.3 (2.7–3.9)
Lipid-lowering medication, %	535 (22.2)	12 540 (11.9)
Height, m	1.68 (1.62–1.75)	1.71 (1.65–1.78)
BMI, kg/m^2^	26 (23–30)	26 (23–28)
Physical inactivity, %	1 602 (67.7)	50 419 (47.9)
Alcohol, items/week	9 (3–18)	8 (4–15)
No. of current smokers, %	1 064 (44.1)	17 463 (16.5)
No. of former smokers, %	1 160 (48.1)	43 052 (40.8)
No. of never smokers, %	188 (7.8)	45 124 (42.7)
Cumulative smoking, pack-years, %	38 (24–50)	15 (6–30)
FEV_1,_ % predicted	61 (46–75)	96 (87–106)
FVC, % predicted	78 (65–92)	100 (90–110)

Values are number (%) for categorical variables and median (interquartile range) for continuous variables. LDL cholesterol was multiplied by 1.43 in participants who reported receiving lipid-lowering medication. Values are from day of enrolment in the Copenhagen General Population Study and onwards. Cumulative smoking values are only from current and former smokers. FEV_1_: Forced expiratory volume in the first second; FVC: Forced vital capacity.

### Genetically observed risk by weighted allele score

**Fig. 2:**
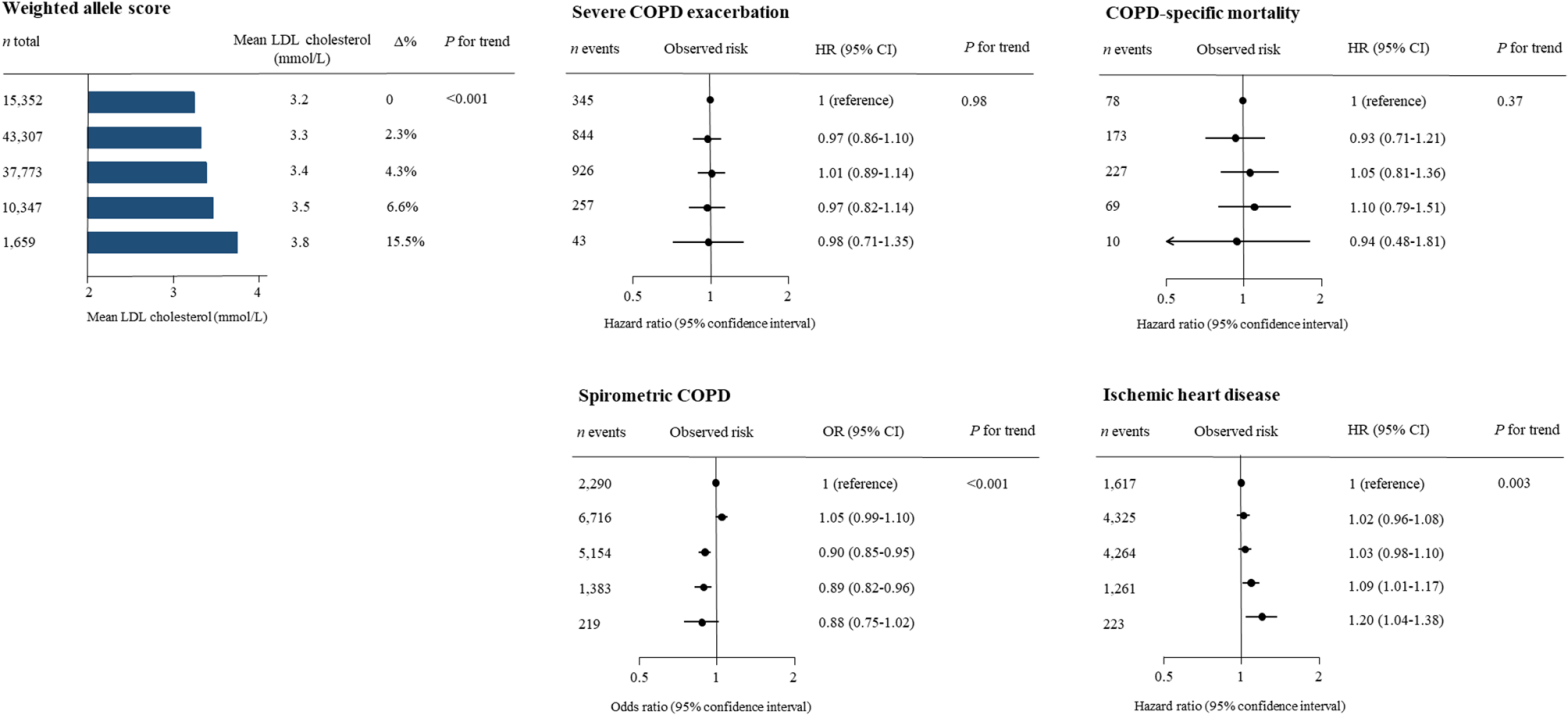
Observed risk of COPD outcomes by LDL cholesterol weighted allele score groups. Odds and hazard ratios for observed risk of COPD were calculated using logistic regression and Cox proportional hazard regression adjusted for age and sex in the Copenhagen General Population Study. Risk of ischemic heart disease was included as a positive control.

None of the genetic variants deviated from the Hardy-Weinberg expectation (*p* values > 0.05). There was a stepwise increase in LDL cholesterol with increasing weighted allele score with a 15.5% increase in the highest group (mean LDL cholesterol: 3.8 mmol/L [147 mg/dL]) compared to the lowest group (3.2 mmol/L [124 mg/dL]) (Fig. 2, upper left corner). All alleles included in the weighted allele score increased LDL cholesterol separately (Supplementary Fig. 1, left panel), fulfilling the relevance assumption (Fig. 1, #1).

There was no statistically significant difference in risk of severe COPD exacerbation (*p* for trend = 0.98) or COPD-specific mortality (*p* for trend = 0.37) between the weighted allele score groups (Fig. 2, upper right panels). Fine and Gray risk estimates with death as competing risk showed similar results for severe COPD exacerbation and COPD-specific mortality (Supplementary Fig. 2). Higher levels of genetic LDL cholesterol were associated with a decreased risk of spirometric COPD with an odds ratio (OR) of 0.89 (95% confidence interval [CI]: 0.82–0.96) for fourth versus first weighted allele score group (*p* for trend < 0.001) (Fig. 2). Risk estimate for ischemic heart disease as the positive control for fifth versus first weighted allele score group was a hazard ratio (HR) of 1.20 (95% CI: 1.04–1.38). Results were similar when stratified for smoking status (Supplementary Fig. 3).

### Potential confounders

**Fig. 3:**
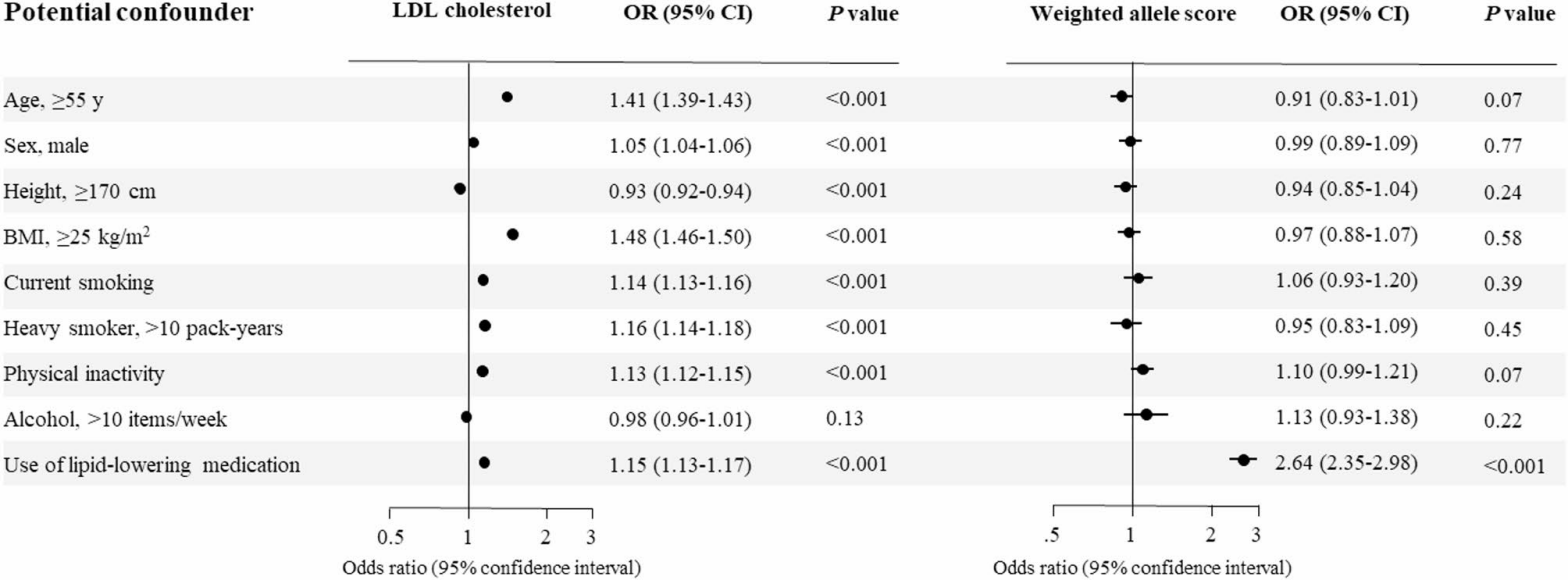
Association of potential confounders with observational LDL cholesterol and weighted allele score. Potential confounders were dichotomized in order to use logistic regression to calculate odd ratios and *p* values.

Potential confounders were mostly associated with LDL cholesterol observationally, but not with the weighted allele score for LDL cholesterol after correction for multiple testing (Fig. 3). Alcohol consumption (more than 10 units per week) was not associated with either LDL cholesterol observationally or genetically. Therefore, confounding or population stratification, as indicated by these covariates, seem improbable, corresponding to the independence assumption (Fig. 1, #2). Use of lipid-lowering medication was as expected associated with the weighted allele score, as elevated LDL cholesterol is one of the main indications for receiving lipid-lowering medication.

### Causal estimates

**Fig. 4:**
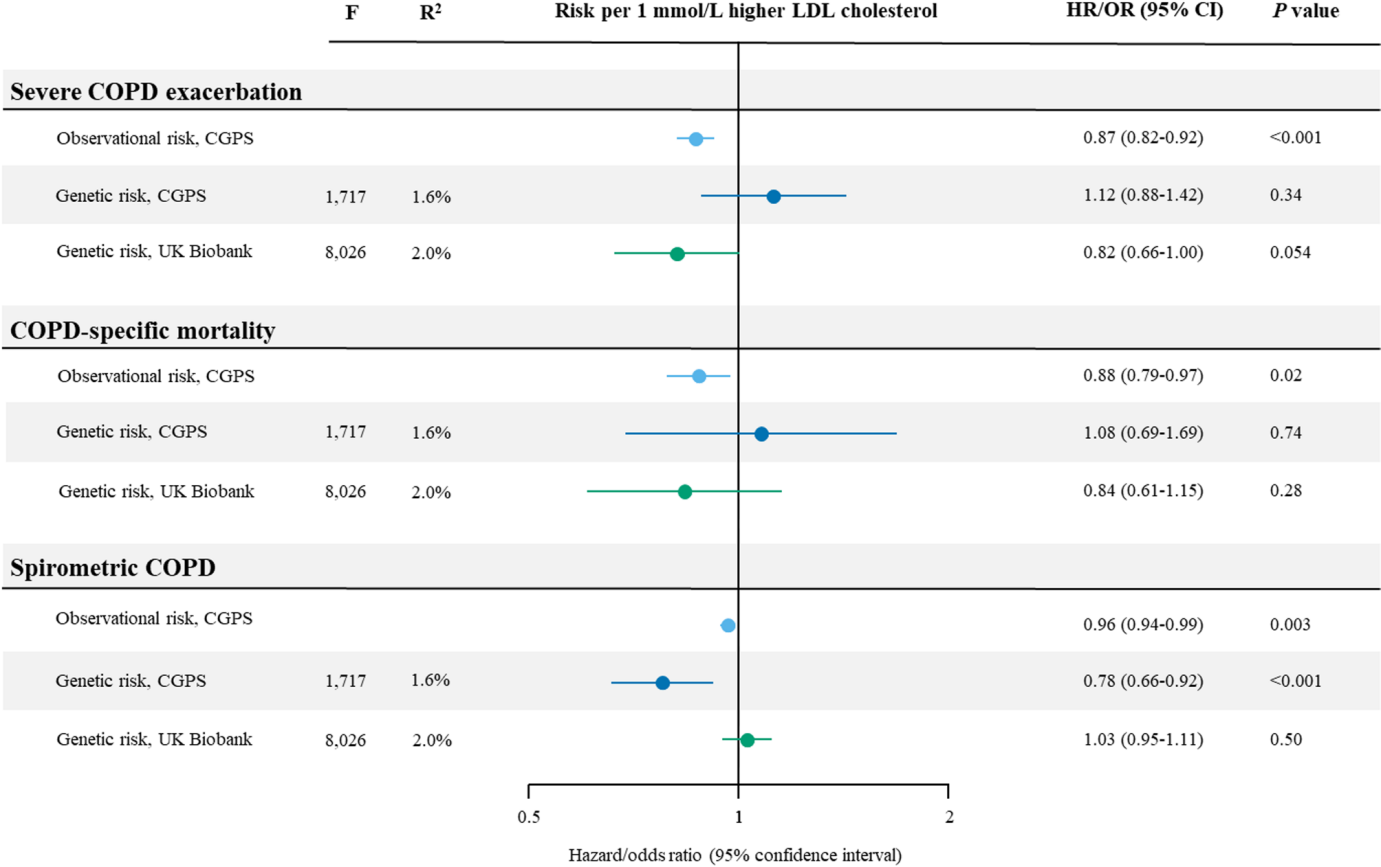
Risk of COPD outcomes per 1 mmol/L higher observational and genetically LDL cholesterol. The hazard and odds ratio for observational LDL cholesterol were calculated using unadjusted Cox proportional hazard regression and logistic regression in the Copenhagen General Population Study (CGPS). Odds ratios for genetically high LDL cholesterol were calculated using individual-level data by generalized method of moments instrumental variable analysis in the CGPS and UK Biobank. F: the strength of the genetic instrument; R^2^: percent contribution of genetic instrument to the variation in LDL cholesterol.

Causal risk estimates for a 1 mmol/L (39 mg/dL) higher LDL cholesterol are shown in Fig. 4. Causal estimates from the CGPS for severe COPD exacerbation (OR 1.12 [95% CI: 0.88–1.42], *p* = 0.34) and COPD-specific mortality (OR 1.08 [0.69–1.69], *p* = 0.74) were both not statistically significant. Similar results were found in the UK Biobank; OR 0.82 (0.66-1.00, *p* = 0.054) for severe COPD exacerbation and 0.84 (0.61–1.15, *p* = 0.28) for COPD-specific mortality. For spirometric COPD, causal estimates from the CGPS found a decreased risk for a 1 mmol/L higher LDL cholesterol (OR 0.78 [0.66–0.92], *p* < 0.001); however, these findings were not reproduced in the UK Biobank (OR 1.03 [0.95–1.11], *p* = 0.50). Individuals with spirometric COPD in UK Biobank were on average younger, had higher LDL cholesterol, lower use of lipid lowering medication, and were less likely to be smokers compared to individuals with spirometric COPD in the CGPS, and more individuals in UK Biobank had self-reported asthma than in the CGPS (Supplementary Table 5). Using alternative calculated weighted allele scores for causal estimates in both the CGPS and UK Biobank to minimize bias or increase power, respectively, results remained similar (Supplementary Fig. 4, Supplementary Tables 6–7). In sensitivity analyses using MR Egger, IVW, weighted median, and weighted mode methods, results were likewise null findings for all three COPD outcomes after correction for multiple testing(Supplementary Fig. 5). Further, MR Egger analyses did not indicate horizontal pleiotropy, as intercepts were insignificant (*p* > 0.05) for all outcomes in both cohorts (Supplementary Fig. 5), corresponding to the exclusion restriction assumption (Fig. 1, #3). When individuals who reported use of lipid-lowering medication were excluded, causal risks of all three outcomes likewise showed similar results (Supplementary Fig. 6).

### Plasma LDL cholesterol and COPD outcomes

**Fig. 5:**
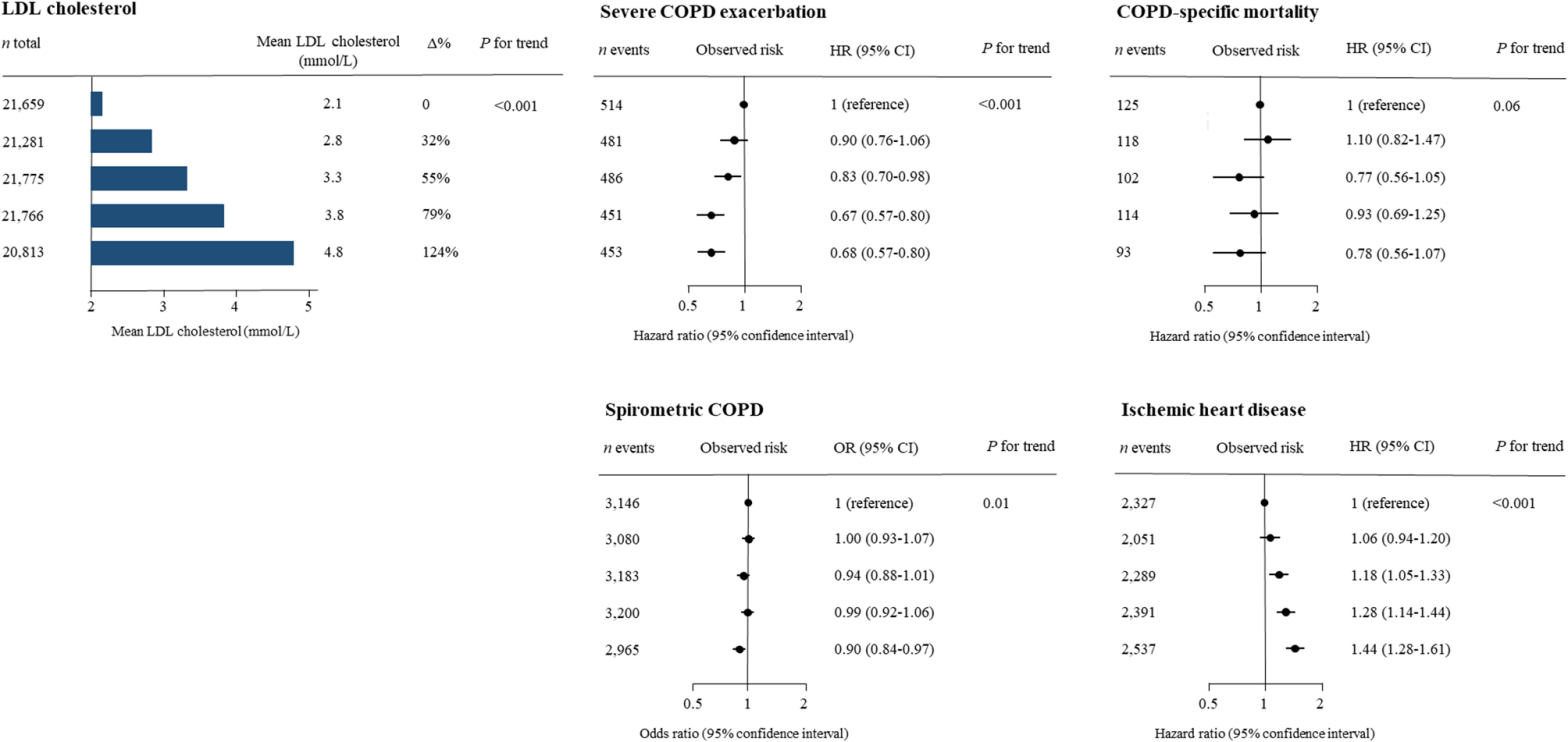
Risk of COPD outcomes by observational LDL cholesterol in quintiles. Odds and hazard ratios for observed risk of COPD were calculated using logistic regression and Cox proportional hazard regression multivariable adjusted for age, sex, BMI, lipid-lowering medication, alcohol consumption, physical inactivity, smoking status, and cumulative smoking in the Copenhagen General Population Study. Risk of ischemic heart disease is included as a positive control.

As shown previously, higher levels of LDL cholesterol were associated with decreased risk of severe COPD exacerbation with a HR of 0.68 (95% CI: 0.57–0.80) for fifth versus first group of LDL cholesterol, and an OR for spirometric COPD of 0.90 (95% CI: 0.84–0.97) for fifth versus first group of LDL cholesterol (Fig. 5). There was no statistically significant difference in HR of COPD-specific mortality by LDL cholesterol quintiles (*p* for trend = 0.06). Risk of ischemic heart disease was included as a positive control with a HR of 1.44 (95% CI: 1.28–1.61) for fifth versus first quintile of LDL cholesterol.

## Discussion

In this one-sample MR study using individual-level data of 108,438 individuals from the CGPS and 389,627 in the UK Biobank, we found that high LDL cholesterol does not causally increase the risk of severe COPD exacerbation and COPD-specific mortality. These results indicate a lack of a protective effect on COPD by lowering LDL cholesterol. High LDL cholesterol was found to decrease the risk of COPD defined by spirometry cross-sectionally in the CGPS but not in the UK Biobank. In sensitivity analyses using MR Egger, IVW, weighted median, and weighted mode methods, higher LDL cholesterol did not increase risk of COPD in any of the three outcomes in either cohort after correction for multiple testing. We believe that the decrease in risk could be a chance finding, as the result was not replicated in the UK Biobank or sensitivity analyses, and does not correspond to the prospective outcomes.

Nevertheless, inter-cohort differences in individuals with spirometric COPD could potentially explain at least part of the differences in results. In the UK Biobank, individuals with spirometric COPD were overall more likely to have self-reported asthma (6.0% in the CGPS vs. 11.5% in UK Biobank), be younger (66 years median in the CGPS vs. 61 years median in the UK Biobank), and smoke less (28.3% never smokers in the CGPS vs. 41.0% never smokers in UK Biobank), indicating that the two spirometric COPD populations might not be comparable (Supplementary Table 5). These differences might also reflect a higher degree of healthy volunteer bias in the UK Biobank, possibly driving results toward the null, as fewer accepted the invitation to participate; in the CGPS 45% of the invited from 2003 to 2019 chose to participate (data not published), whereas only 5.5% of invited between 2006 and 2010 participated in the UK Biobank^31^. In any case, if the difference in spirometric COPD results was due to differences in characteristics or bias between the cohorts, we would expect results of the sensitivity analyses to correspond to the primary MR analyses, which they do not. Further, null findings in an MR study are likely to reflect a true null relationship, as bias from possible pleiotropy is more likely to lead to false positive rather than false negative findings^32,33^. It is also important to note that spirometric COPD was the only cross-sectional outcome and, therefore, a weaker outcome for ascertaining causality. Emphasis should consequently be put on the two prospective outcomes, severe COPD exacerbation and COPD-specific death, which are more clinically relevant and were unequivocally null.

We previously found that low LDL cholesterol was observationally associated with an increased risk of severe COPD exacerbation and COPD-specific mortality^12^. These findings were attributed to reverse causality, which the negative findings of this causal one-sample MR study support. Previous MR studies investigating LDL cholesterol targets and COPD have found contradictory results, concluding either no causal relationship^34^, causal association between *LDLR* (but not *APOB* and *PCSK9*) and COPD^35^, or causal association between *PCSK9* and *HMGCR* (but not *APOB*, *LDLR*, or *NPC1L1*) and COPD^36^. Importantly, these MR studies all relied solely on two-sample MR methodology, which has been critiqued to add little, if any, scientific value, and advised to be rejected by peer-reviewers^37^. Of interest, another individual-level data MR study investigating associations solely between *PCSK9* variants and a large range of outcomes likewise did not find a causal association with COPD^38^. Observational studies and randomized controlled trials (RCTs) have found contradictory results regarding the effect of statins on COPD. A recent review of observational studies investigating the effects of statins on mortality in individuals with COPD found a reduced risk of mortality (pooled relative risk 0.66 (95% CI: 0.59–0.72)), though they concluded that the included studies were affected by major biases^8^. Also, a Cochrane review from 2019 on RCTs investigating statins’ effects on COPD found improvement in FEV_1_/FVC (mean difference 2.66% (95% CI: 0.12–5.2, *p* = 0.04) based on six studies^9,10,39–42^. Further, they found no statistically significant difference in number of exacerbations per person-year and COPD-specific mortality. Both of these outcomes were, however, only investigated in one included RCT, namely STATCOPE^43^. In 2021, a second major RCT by Schenk et al. investigated the effect of statins on COPD exacerbation, and found a 23% relative reduction in annualised exacerbation, and a HR of 0.51 (95% CI: 0.34–0.75) for risk of first exacerbation^5^.

These effects of statins on COPD have been attributed to pleiotropic effects, though this has not been established definitively^7–10^. In addition to its’ LDL cholesterol lowering effects, statins have shown anti-inflammatory properties through reduction of CRP and inhibiting recruitment of inflammatory cells, as well as anti-oxidative properties by inducing cellular accumulation of nitric oxide synthase^44^. As the present study found no causal association between LDL cholesterol and COPD, and therefore a lack of a protective effect of lowering LDL cholesterol, these results indirectly support a possible effect through statins’ pleiotropic pathways. Further studies investigating concrete mechanisms of the effect of statins on COPD exacerbations are needed.

As baseline measurements of LDL cholesterol were used in this study, the effect of intra-individual variability of LDL cholesterol over time on COPD could not be evaluated. High variability in LDL cholesterol has been associated with increased risk of cardiovascular disease and all-cause mortality^45^, and variability in other biomarkers, such as eosinophils, has shown to be increased in individuals with COPD^46^. However, as our results indicate that levels of LDL cholesterol do not have an effect on COPD outcomes, it seems unlikely that differences in intra-individual variability in LDL cholesterol or extreme LDL cholesterol levels should have an effect.

Our study has several strengths. First, as the study design is MR, causal risk estimates can be determined with more confidence than in other forms of epidemiological research. Second, in this MR study, we rely on one-sample individual-level data replicated in a second cohort, which highly increases the scientific value of the results compared to two-sample MR^37^. Third, all participants in the CGPS were randomly selected from the general population with a relatively high inclusion rate (45%), thereby minimizing the risk of bias from healthy participant selection. Fourth, the prospective outcomes of severe COPD exacerbation and COPD-specific mortality were obtained from complete national Danish health registries, having minimal loss to follow-up, and results for these two prospective outcomes were consistent across multiple weighted allele scores, sensitivity analyses, and in the UK Biobank, increasing the generalisability of the findings.

Limitations of this study include that postbronchodilator spirometry measurements were not available, which might falsely categorise some individuals as having COPD defined by spirometry rather than asthma. To account for this, individuals reporting that they have asthma were excluded from these estimates, as done previously^12,47^. Second, causal estimates in the primary analysis for spirometric COPD were different in the two cohorts. We believe that the decreased risk of spirometric COPD in the primary instrumental variable analysis in the CGPS could represent a chance finding without relation to any actual biological process as this finding was not replicated in sensitivity analyses, nor the UK Biobank; however, given the results in our paper, we naturally cannot completely exclude that high LDL cholesterol could drive lower risk of COPD. Third, only individuals of white European ancestry were included, and results might not be transferable to other ethnicities, though, to the best of our knowledge, there is no information suggesting that results are not representative of other populations. Fourth, smoking status and history of smoking were self-reported, which is likely to be influenced by recall bias and underreporting, though it is believed that recall would be equal between smokers with and without COPD^48,49^. Fifthly, though F-statistics values of the genetic instruments are extremely high, weak instrument bias might still be present, pulling MR results towards the observational findings as R^2^ is 1.6% for the primary instrument used in the CGPS. Lastly, a bidirectional MR analysis was not performed as genetic variants associated with COPD were not available in the CGPS, and since this was outside the scope of the study.

## Conclusion

In this one-sample MR study using individual-level data and two large cohorts, we found that high LDL cholesterol did not causally increase the risk of COPD. Our results indicate that a protective effect of statins on COPD exacerbation and COPD-specific mortality cannot be attributed to LDL cholesterol-lowering, which supports the possibility that pleiotropic effects of statins may rather be responsible. However, inconsistency observed in cross-sectional analyses suggests that further validation is warranted.

## Supplementary Information

Below is the link to the electronic supplementary material.

Supplementary Material 1

## Data Availability

The datasets supporting the conclusions of this article were used under license and cannot be made publicly available in a repository due to privacy regulations. Access to data from the UK Biobank is available for *bona fide* researchers with permission to the UK Biobank. For further information on gaining access, please see [https://www.ukbiobank.ac.uk/enable-your-research](https:/www.ukbiobank.ac.uk/enable-your-research) .

## Abbreviations

BMI: Body mass index
CI: Confidence interval
COPD: Chronic obstructive pulmonary disease
CPGS: Copenhagen General Population Study
FEV_1_: Forced expiratory volume in the first second
FVC: Forced vital capacity
GWAS: genome-wide association study
HR: Hazard ratio
ICD: International Classification of Diseases
IVW: Inverse-variance weighted
LDL: Low-density lipoprotein
MR: Mendelian randomization
OR: Odds ratio
RCT: Randomized controlled trials
SNP: single nucleotide polymorphism
UKB: UK Biobank
