# Supplementary material for: Low-density lipoprotein cholesterol and risk of COPD: a Mendelian randomization study
Source: Sci Rep. 2026 Apr 15;16:17485. doi: 10.1038/s41598-026-48823-6 (PMC13237049; doi:10.1038/s41598-026-48823-6)
Supplement: Supplementary file 1 — Supplementary Material 1 [file 41598_2026_48823_MOESM1_ESM.docx]

**Supplementary**

Low-density lipoprotein cholesterol and risk of COPD: A Mendelian Randomization Study

Justesen JF, Wadström BN, Marott SCW, Landt EM, Nordestgaard BG, Afzal S, Dahl M

**Table of Contents**

[Appendix 1. Methods: Copenhagen General Population Study 2](#_Toc218927930)

[Weighted allele score CGPS 2 2](#_Toc218927931)

[Appendix 2. Methods: UK Biobank variables 2](#_Toc218927932)

[Weighted allele score UK Biobank 2 2](#_Toc218927933)

[Appendix 3. Methods: Supplementary Figures 2](#_Toc218927934)

[Supplementary Table 1. 4](#_Toc218927935)

[Supplementary Table 2 5](#_Toc218927936)

[Supplementary Table 3. 6](#_Toc218927937)

[Supplementary Table 4. 11](#_Toc218927938)

[Supplementary Figure 1. 12](#_Toc218927939)

[Supplementary Figure 2. 13](#_Toc218927940)

[Supplementary Figure 3. 14](#_Toc218927941)

[Supplementary Table 5 15](#_Toc218927942)

[Supplementary Figure 4. 16](#_Toc218927943)

[Supplementary Table 6. 17](#_Toc218927944)

[Supplementary Table 7. 18](#_Toc218927945)

[Supplementary Figure 5. 25](#_Toc218927946)

[Supplementary Figure 6. 26](#_Toc218927947)

## Appendix 1. Methods: Copenhagen General Population Study

### Weighted allele score CGPS 2

To minimize bias from statin use and the risk of overfitting, an alternative weighted allele score was calculated in the CGPS for causal analyses, as seen in Supplementary Figure 4. The weighted allele score CGPS 2 was calculated similarly to the primary CGPS weighted allele score, however *β*-coefficients were estimated solely from individuals under 50 years of age. These *β*-coefficients were then used to calculate a weighted allele score for each participant. *LDLR W556S* was omitted from the weighted allele score because of collinearity. See Supplementary Table 6 for *β*-coefficients and standard errors.

## Appendix 2. Methods: UK Biobank variables

### Weighted allele score UK Biobank 2

Genetic variants from the entire autosome associated with LDL cholesterol at *p*<5 x 10^-8^ and R^2^<0.001 in the same previous GWAS were included in this weighted allele score (1). By including variants from the entire autosome, the weighted allele score gains a higher percentual contribution of the variation in LDL cholesterol (6.9%), though there might be introduced more bias (2). All included genetic variants and their *β*-coefficients can be seen in Supplementary Table 7.

## Appendix 3. Methods: Supplementary Figures

Subgroup analyses of observed risk of COPD for LDL cholesterol were performed for LDL cholesterol increasing alleles separately (Supplementary Figure 1) and smoking status (Supplementary Figure 3). Cox proportional hazards regression and logistic regression, both adjusted for sex and age, were used to calculate estimates. In subgroups of LDL cholesterol alleles separately (Supplementary Figure 1), *APOB* and *LDLR* alleles were combined to ensure an adequate number of events and *PCSK9* alleles were combined and divided into groups according to number of LDL cholesterol increasing alleles. Causal estimates of risk of COPD per 1 mmol/L (39 mg/dL) higher LDL cholesterol for the different weighted allele scores in CGPS and UK Biobank (Supplementary Figure 4) or with individuals who reported use of lipid-lowering medication in the CGPS (Supplementary Figure 6) were estimated using the *ivpoisson* command in Stata. In the UK Biobank *ivpoisson* were adjusted for the 10 first principal components and genotype chip, as in Figure 4.

**References**

1. Wadström BN, Borges MC, Wulff AB, et al. Elevated Remnant and LDL Cholesterol and the Risk of Peripheral Artery Disease: A Mendelian Randomization Study. J Am Coll Cardiol. 2025 Apr;85(12):1353–68.

2. Burgess S, Thompson SG. Use of allele scores as instrumental variables for Mendelian randomization. Int J Epidemiol. 2013 Aug;42(4):1134–44.

## Supplementary Table 1.

| **Cohort** | *n* | **Exposure** | **Instrument** | **Outcomes** | | |
| --- | --- | --- | --- | --- | --- | --- |
|  |  | **LDL cholesterol** | **Weighted allele score** | **Severe COPD exacerbation** | **COPD-specific mortality** | **Spirometric COPD** |
| **The Copenhagen General Population Study** | 108 438 | Calculated by the Friedewald equation when triglycerides ≤4 mmol/L. Measured directly when triglycerides >4 mmol/L | 9 variants in biological relevant genes *APOB*, *LDLR*, *PCSK9*, *HMGCR*, and *NPC1L1*.  See Sup. Table 2. | Primary diagnosis of ICD10 J44  *or*  secondary diagnosis of ICD10 J44 and either primary diagnosis of J96 or J18. | ICD10 J41-44 as primary cause of death | FEV_1_/FVC <0.7.  Self-reported asthma diagnosis excluded. |
| **The UK Biobank** | 389 627 | Measured directly (>0 mmol/L)  (Data-Field 30780) | 63 variants in proximity (+/- 100 kb) of biological relevant genes *APOB*, *LDLR*, *PCSK9*, *HMGCR*, and *NPC1L1* from previous GWAS (Wadström et al. J Am Coll Cardiol. 2025)  See Sup. Table 3. | Primary diagnosis of ICD10 J44  *or*  secondary diagnosis of ICD10 J44 and either primary diagnosis of J96 or J18. | ICD10 J41-44 as primary cause of death | FEV_1_/FVC <0.7.  (Data_Field 3063, 3062)  Self-reported asthma diagnosis excluded.  (Data_Field 6152) |

Description of number individuals included, exposure (*n*), genetic instrument, and outcomes used in the Copenhagen General Population Study and UK Biobank. ICD: International Classification of Diseases.

Supplementary Table 2**.**

| **Gene** | **rs number** | **Effect allele** | **Other allele** | **β-coefficient** | **SE** | ***P* value** |
| --- | --- | --- | --- | --- | --- | --- |
| *APOB 3500Q* | rs5742904 | A | G | 1.91222 | 0.1136317 | 2 x 10^-63^ |
| *LDLR W23X* | rs267607213 | A | G | 2.629312 | 0.387913 | 1 x 10^-11^ |
| *LDLR W66G* | rs121908025 | G | T | 2.733412 | 0.1706978 | 1 x 10^-57^ |
| *LDLR W556S* | rs138947766 | C | G | 1.697721 | 0.9504347 | 0.074 |
| *PCSK9 R46L* | rs11591147 | T | G | -0.3880492 | 0.023898 | 3 x 10^-59^ |
| *PCSK9 V474I* | rs562556 | G | A | -0.0409488 | 0.0074863 | 5 x 10^-08^ |
| *PCSK9 E670G* | rs505151 | G | A | 0.0808741 | 0.0144309 | 2 x 10^-08^ |
| *HMGCR* | rs17238484 | T | G | 0.0610986 | 0.0064744 | 4 x 10^-21^ |
| *NPC1L1* | rs41279633 | T | C | 0.05101 | 0.0072443 | 2 x 10^-12^ |

Weights per allele used for weighted allele score in the Copenhagen General Population Study seen in Figure 4. SE = standard error.

## Supplementary Table 3.

|  | **rs number** | **Effect allele** | **Other allele** | **β-coefficient** | **SE** | ***P* value** |
| --- | --- | --- | --- | --- | --- | --- |
| 1 | rs34232196 | C | T | 0.0559362 | 0.00391436 | 3 x 10^-46^ |
| 2 | rs17111503 | A | G | -0.0418324 | 0.0038083 | 5 x 10^-28^ |
| 3 | rs11591147 | G | T | 0.361227 | 0.0127876 | 2 x 10^-175^ |
| 4 | rs472495 | G | T | -0.047615 | 0.00352254 | 1 x 10^-41^ |
| 5 | rs77875082 | G | A | -0.0580189 | 0.00971563 | 2 x 10^-09^ |
| 6 | rs374459115 | G | A | -0.0688518 | 0.0149771 | 4 x 10^-06^ |
| 7 | rs2479420 | C | T | 0.0293297 | 0.00384925 | 3 x 10^-14^ |
| 8 | rs12739979 | C | T | 0.019233 | 0.0040996 | 3 x 10^-06^ |
| 9 | rs72660548 | C | G | -0.0673631 | 0.0124163 | 6 x 10^-08^ |
| 10 | rs10888896 | G | C | -0.0249542 | 0.0038945 | 2 x 10^-10^ |
| 11 | rs557211 | G | T | -0.0209652 | 0.00439896 | 2 x 10^-06^ |
| 12 | rs45613943 | T | C | 0.0417131 | 0.00791035 | 1 x 10^-07^ |
| 13 | rs150119739 | G | A | -0.0635063 | 0.0083969 | 4 x 10^-14^ |
| 14 | rs7525503 | G | T | -0.064334 | 0.0125092 | 3 x 10^-07^ |
| 15 | rs505151 | G | A | 0.0869339 | 0.00958222 | 1 x 10^-19^ |
| 16 | rs4927194 | C | T | 0.0626075 | 0.0107404 | 6 x 10^-09^ |
| 17 | rs12916 | T | C | -0.0649484 | 0.00340034 | 3 x 10^-81^ |
| 18 | rs111970624 | G | A | -0.051729 | 0.00690581 | 7 x 10^-14^ |
| 19 | rs4703665 | T | C | -0.0227357 | 0.00474163 | 2 x 10^-06^ |
| 20 | rs115664150 | C | T | -0.0387683 | 0.00678136 | 1 x 10^-08^ |
| 21 | rs144083983 | C | T | 0.0360142 | 0.00625437 | 9 x 10^-09^ |
| 22 | rs151000110 | G | A | -0.0558306 | 0.00715737 | 6 x 10^-15^ |
| 23 | rs73013176 | T | C | 0.182478 | 0.0162836 | 4 x 10^-29^ |
| 24 | rs112634605 | G | C | 0.10025 | 0.00939756 | 1 x 10^-26^ |
| 25 | rs143020224 | C | G | 0.181117 | 0.00535957 | 1 x 10^-200^ |
| 26 | rs6511721 | G | A | 0.0437426 | 0.00345553 | 1 x 10^-36^ |
| 27 | rs145790091 | T | A | 0.0657579 | 0.00361045 | 4 x 10^-74^ |
| 28 | rs146576912 | C | T | 0.119008 | 0.00803104 | 1 x 10^-49^ |
| 29 | rs55935710 | C | T | 0.0252238 | 0.00356511 | 2 x 10^-12^ |
| 30 | rs36005514 | G | A | -0.0440341 | 0.00665456 | 4 x 10^-11^ |
| 31 | rs17248748 | C | T | 0.0769129 | 0.0137743 | 2 x 10^-08^ |
| 32 | rs3745677 | G | A | -0.0590636 | 0.007257 | 4 x 10^-16^ |
| 33 | rs12611153 | C | T | 0.0404145 | 0.00417357 | 4 x 10^-22^ |
| 34 | rs116959285 | C | G | -0.0731118 | 0.00878209 | 8 x 10^-17^ |
| 35 | rs7247905 | G | A | 0.066326 | 0.010663 | 5 x 10^-10^ |
| 36 | rs17249001 | G | A | -0.0514677 | 0.00630658 | 3 x 10^-16^ |
| 37 | rs3826810 | G | A | -0.0444123 | 0.00848625 | 2 x 10^-07^ |
| 38 | rs140181075 | C | T | -0.0494185 | 0.00923975 | 9 x 10^-08^ |
| 39 | rs117339792 | G | A | 0.0857795 | 0.0135116 | 2 x 10^-10^ |
| 40 | rs4804149 | T | C | -0.0222316 | 0.00380118 | 5 x 10^-09^ |
| 41 | rs379309 | C | T | 0.0189411 | 0.003462 | 5 x 10^-08^ |
| 42 | rs17699030 | A | G | 0.0492836 | 0.0096187 | 3 x 10^-07^ |
| 43 | rs41279633 | G | T | -0.0462563 | 0.00459113 | 7 x 10^-24^ |
| 44 | rs217399 | C | T | 0.0228681 | 0.00336847 | 1 x 10^-11^ |
| 45 | rs10198175 | A | G | 0.0502246 | 0.00537831 | 10 x 10^-21^ |
| 46 | rs6732011 | G | T | 0.0383985 | 0.0035507 | 3 x 10^-27^ |
| 47 | rs1317821 | C | T | -0.0488652 | 0.00384192 | 5 x 10^-37^ |
| 48 | rs533617 | T | C | 0.134047 | 0.00859545 | 8 x 10^-55^ |
| 49 | rs9282606 | G | T | -0.0746983 | 0.00784662 | 2 x 10^-21^ |
| 50 | rs934197 | G | A | -0.0854791 | 0.00353384 | 3 x 10^-129^ |
| 51 | rs562338 | A | G | -0.108021 | 0.00437318 | 1 x 10^-134^ |
| 52 | rs62120800 | G | A | 0.0868916 | 0.0172478 | 5 x 10^-07^ |
| 53 | rs80169634 | G | A | 0.0428796 | 0.00784785 | 5 x 10^-08^ |
| 54 | rs4507059 | A | G | 0.0261609 | 0.0036562 | 8 x 10^-13^ |
| 55 | rs576306544 | C | A | 0.0748961 | 0.0154536 | 1 x 10^-06^ |
| 56 | rs1042023 | G | C | -0.110519 | 0.0169082 | 6 x 10^-11^ |
| 57 | rs150856817 | T | A | 0.0563893 | 0.00650316 | 4 x 10^-18^ |
| 58 | rs35750610 | T | C | -0.0288358 | 0.00561675 | 3 x 10^-07^ |
| 59 | rs72902594 | C | A | 0.0607483 | 0.00699842 | 4 x 10^-18^ |
| 60 | rs6756743 | C | T | -0.0372575 | 0.00788846 | 2 x 10^-06^ |
| 61 | rs145449541 | G | A | 0.092453 | 0.0162328 | 1 x 10^-08^ |
| 62 | rs114185526 | C | T | 0.0990307 | 0.015952 | 5 x 10^-10^ |
| 63 | rs548774896 | T | G | -0.0254221 | 0.00531924 | 2 x 10^-06^ |

Information on alleles used in UK Biobank weighted allele score seen in Figure 4. SE = standard error.

## Supplementary Table 4.

| **Instrumental variable assumptions** | **The Copenhagen General**  **Population Study** | **The UK Biobank** |
| --- | --- | --- |
| **Assumption 1**  The relevance assumption | 9 genetic variants chosen from prespecified genes with known roles in the LDL metabolism (*APOB*, *PCSK9*, *LDLR*, *HMGCR*, and *NPC1L1)* and previously used.  F-value = 1,717, R^2^ = 1.6%. | 63 genetic variants chosen from prespecified genes with known roles in the LDL metabolism and   1. in the proximity (+/- 100 kb) of the *APOB*, *PCSK9*, *LDLR*, *HMGCR*, and *NPC1L1* genes 2. had R^2^<0.1 3. were associated with LDL cholesterol at *p*<5 x 10^-6^ in a previous GWAS in individuals <50 years old in the UK Biobank (Wadström et al. J Am Coll Cardiol. 2025)   F-value = 8,026, R^2^ = 2.02%. |
| **Assumption 2**  The independence assumption | There was no association between the weighted allele score and potential confounders except use of lipid-lowering medication as expected, see Figure 3. | Association between the weighted allele score and potential confounders previously thoroughly investigated with no association except, apolipoprotein B, triglycerides, and use of lipid-lowering medication as expected (Wadström et al. J Am Coll Cardiol. 2025). |
| **Assumption 3**  The exclusion restriction assumption | Horizontal pleiotropy deemed unlikely as genetic variants were chosen in biologically relevant genes. MR Egger, weighted median, and weighted mode methods were performed as seen in Supplementary Figure 5. Intercepts of MR Egger were insignificant (*p*>0.05) for all outcomes in both cohorts. | Horizontal pleiotropy deemed unlikely as genetic variants were chosen in biologically relevant genes. MR Egger, weighted median, and weighted mode methods were performed as seen in Supplementary Figure 5. Intercepts of MR Egger were insignificant (*p*>0.05) for all outcomes in both cohorts. |

Instrumental variable assumption and assessment of the genetic instruments applied in the Copenhagen General Population Study and UK Biobank. F: the strength of the genetic instrument; R^2^: percent contribution of genetic instrument to the variation in LDL cholesterol.

## Supplementary Figure 1.


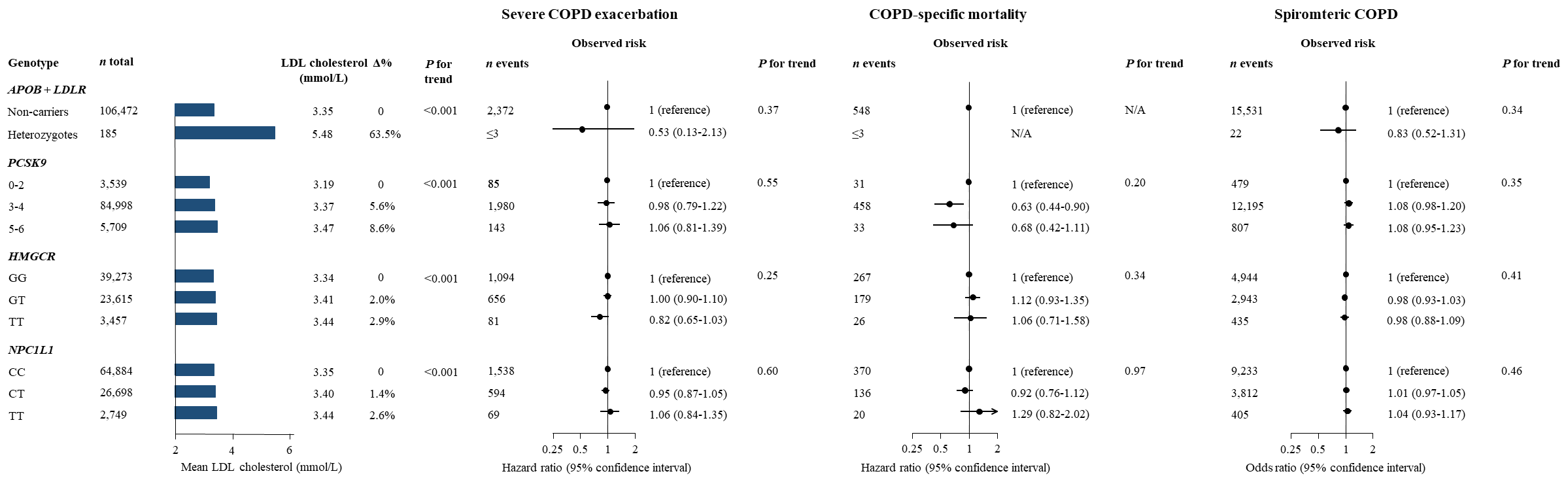


Observed risk of COPD outcomes as a function of LDL cholesterol increasing alleles separately in the Copenhagen General Population Study. *APOB* and *LDLR* alleles were combined. *PCSK9* alleles were combined and divided into groups according to number of LDL cholesterol increasing alleles. Cox proportional hazard regression and logistic regression both adjusted for age and sex were used for risk estimates.

## Supplementary Figure 2.


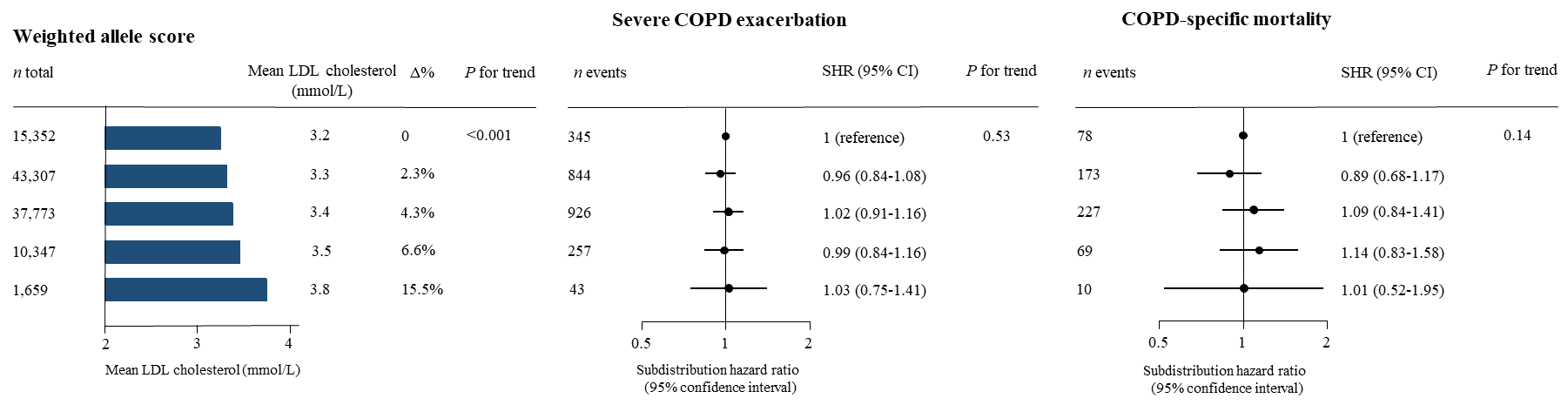


Fine and Gray competing risk of severe COPD exacerbation and COPD-specific mortality with all-cause mortality as competing risk as a function of weighted allele score group in the Copenhagen General Population Study. Risk estimates were adjusted for age and sex.

## **Supplementary Figure 3.**


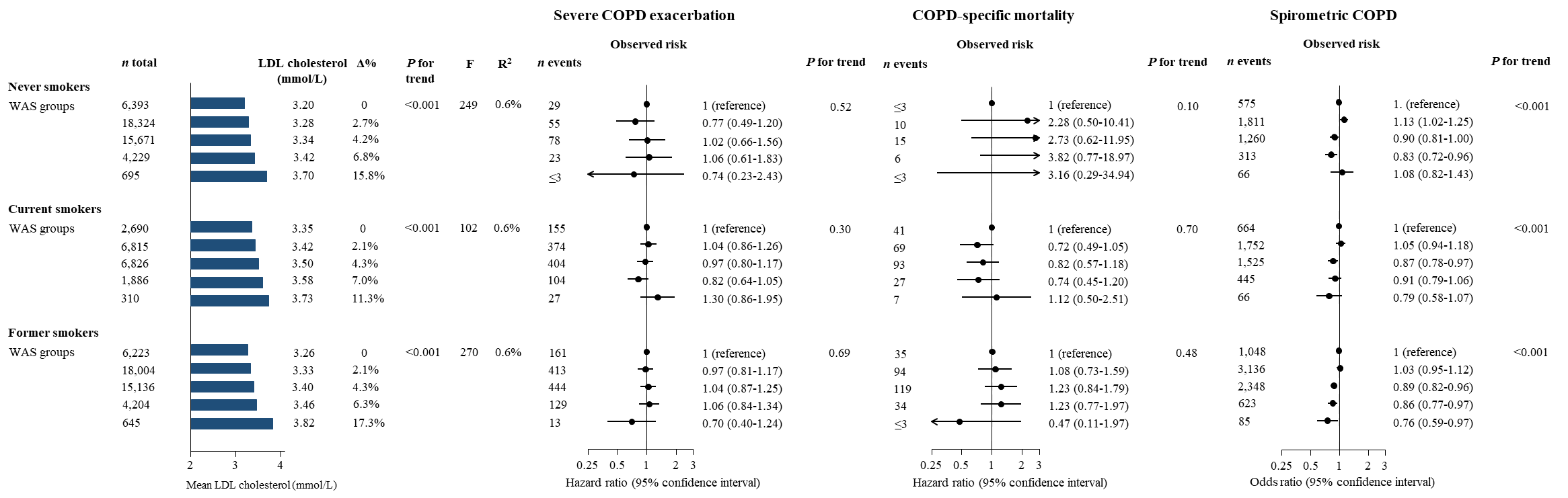


Observed risk of COPD outcomes as a function of weighted allele score group stratified by smoking status in the Copenhagen General Population Study. Cox proportional hazards regression and logistic regression both adjusted for age and sex were used for risk estimates. WAS: weighted allele score.

##

## Supplementary Table 5

|  | **CGPS** | **UK Biobank** |
| --- | --- | --- |
| No. of individuals with spirometric COPD, % of entire cohort | 15 762 (17.0) | 43 494 (12.0) |
| Age, years | 66 (58-74) | 61 (55-65) |
| Women, % | 8 175 (51.9) | 19 433 (44.7) |
| LDL cholesterol, mmol/L | 3.1 (2.5-3.8) | 3.5 (2.9-4.1) |
| Lipid-lowering medication, % | 2 827 (17.9) | 6 281 (14.4) |
| No. of current smokers, % | 4 025 (25.6) | 8 663 (19.9) |
| No. of former smokers, % | 7 240 (46.1) | 16 805 (38.6) |
| No. of never smokers, % | 4 452 (28.3) | 17 837 (41.0) |
| Cumulative smoking, pack-years, % | 26 (12-41) | 27 (15-42) |
| No. of individuals with self-reported asthma in entire cohort, %* | 6 472 (6.0) | 44 874 (11.5) |

Characteristics of individuals with spirometric COPD in the Copenhagen General Population Study (CGPS) and UK Biobank. Values are number (%) for categorical variables and median (interquartile range) for continuous variables. Values are from day of enrolment and onwards. Cumulative smoking values are only from current and former smokers. *Excluded from spirometric COPD, therefore not included in no. of individuals.

## Supplementary Figure 4.


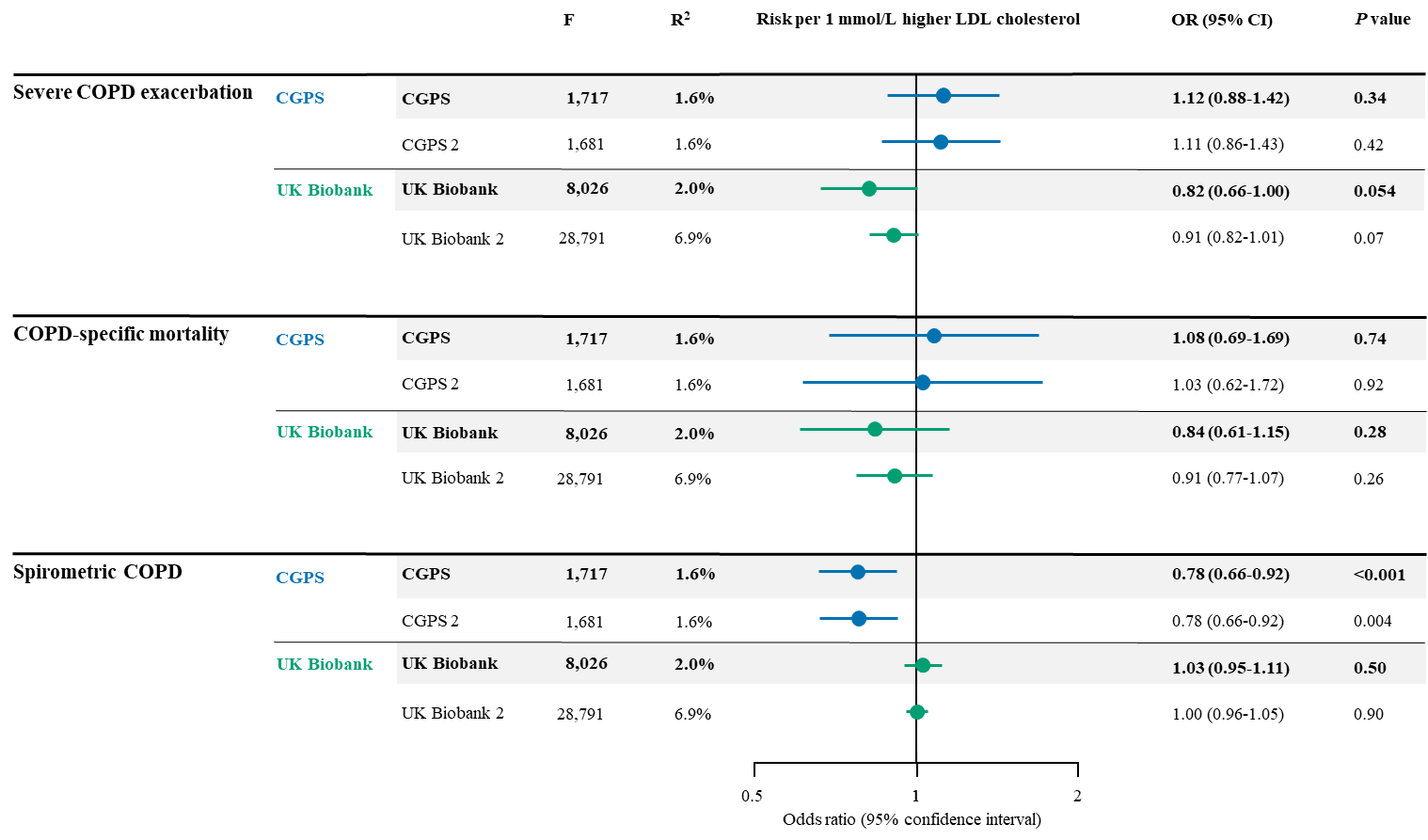


Alternative weighted allele scores for the Copenhagen General Population Study (CGPS) and UK Biobank. Odds ratios for genetically high LDL cholesterol were calculated by generalized method of moments instrumental variable analysis. Highlighted estimates indicate the primary analyses used in the main manuscript. F: the strength of the genetic instrument; R^2^: percent contribution of genetic instrument to the variation in LDL cholesterol. Analyses in the UK Biobank were adjusted for 10 principal components and genotype chip.

Supplementary Table 6.

| **Gene** | **rs number** | **Effect allele** | **Other allele** | **β-coefficient** | **SE** | ***P* value** |
| --- | --- | --- | --- | --- | --- | --- |
| *APOB 3500Q* | rs5742904 | A | G | 2.010192 | 0.1842399 | 1 x 10^-27^ |
| *LDLR W23X* | rs267607213 | A | G | 1.413182 | 0.6377596 | 0.027 |
| *LDLR W66G* | rs121908025 | G | T | 2.682838 | 0.2720106 | 6 x 10^-23^ |
| *LDLR W556S* | rs138947766 | C | G | - | - | - |
| *PCSK9 R46L* | rs11591147 | T | G | -0.4072963 | 0.0414065 | 7 x 10^-23^ |
| *PCSK9 V474I* | rs562556 | G | A | -0.032389 | 0.0127619 | 0.011 |
| *PCSK9 E670G* | rs505151 | G | A | 0.110505 | 0.0243854 | 6 x 10^-06^ |
| *HMGCR* | rs17238484 | T | G | 0.0500401 | 0.0110199 | 6 x 10^-06^ |
| *NPC1L1* | rs41279633 | T | C | 0.0643501 | 0.0123318 | 2 x 10^-07^ |

Weights per allele used for the Copenhagen General Population Study weighted allele score (CGPS 2) used in Supplementary Figure 4. *LDLR W556S* omitted because of collinearity. SE = Standard error.

## Supplementary Table 7.

|  | **rs number** | **Effect allele** | **Other allele** | **β-coefficient** | **SE** | ***P* value** |
| --- | --- | --- | --- | --- | --- | --- |
| 1 | rs10195252 | T | C | 0.0206109 | 0.00341841 | 2 x 10^-09^ |
| 2 | rs10455872 | A | G | -0.0886769 | 0.00611547 | 1 x 10^-47^ |
| 3 | rs10468017 | C | T | -0.0314255 | 0.00364034 | 6 x 10^-18^ |
| 4 | rs1049178 | A | G | -0.0437322 | 0.00460486 | 2 x 10^-21^ |
| 5 | rs11065384 | T | C | 0.033482 | 0.00360464 | 2 x 10^-20^ |
| 6 | rs111409688 | C | T | 0.0252961 | 0.00432992 | 5 x 10^-09^ |
| 7 | rs11206517 | T | G | -0.084492 | 0.00947326 | 5 x 10^-19^ |
| 8 | rs113360274 | A | G | -0.0258185 | 0.00427435 | 2 x 10^-09^ |
| 9 | rs114165349 | G | C | -0.0693746 | 0.0110942 | 4 x 10^-10^ |
| 10 | rs115478735 | A | T | -0.0632209 | 0.0042984 | 6 x 10^-49^ |
| 11 | rs11591147 | G | T | 0.361227 | 0.0127876 | 2 x 10^-175^ |
| 12 | rs117733303 | A | G | -0.114464 | 0.0121845 | 6 x 10^-21^ |
| 13 | rs118170342 | T | C | -0.127011 | 0.00879729 | 3 x 10^-47^ |
| 14 | rs12208357 | C | T | -0.0556263 | 0.00656212 | 2 x 10^-17^ |
| 15 | rs1229984 | T | C | -0.0638028 | 0.0114782 | 3 x 10^-08^ |
| 16 | rs12693971 | T | C | -0.0294281 | 0.00384007 | 2 x 10^-14^ |
| 17 | rs12916 | T | C | -0.0649484 | 0.00340034 | 5 x 10^-81^ |
| 18 | rs12945886 | A | G | 0.0234685 | 0.003968 | 3 x 10^-09^ |
| 19 | rs13108218 | A | G | 0.0285637 | 0.00345542 | 1 x 10^-16^ |
| 20 | rs13255048 | G | A | -0.0268286 | 0.0048435 | 3 x 10^-08^ |
| 21 | rs143020224 | C | G | 0.181117 | 0.00535957 | 1 x 10^-200^ |
| 22 | rs147711004 | G | A | -0.149419 | 0.00932843 | 1 x 10^-57^ |
| 23 | rs148601586 | C | G | -0.137392 | 0.0150581 | 7 x 10^-20^ |
| 24 | rs149394327 | G | C | -0.0799849 | 0.00978848 | 3 x 10^-16^ |
| 25 | rs1500187 | A | G | 0.0202881 | 0.00335037 | 1 x 10^-09^ |
| 26 | rs17050272 | G | A | 0.0244546 | 0.0034035 | 7 x 10^-13^ |
| 27 | rs174564 | A | G | 0.0400232 | 0.00349916 | 3 x 10^-30^ |
| 28 | rs1800961 | C | T | 0.0598392 | 0.00954137 | 4 x 10^-10^ |
| 29 | rs1883711 | G | C | -0.114116 | 0.00965363 | 3 x 10^-32^ |
| 30 | rs2066905 | G | C | 0.0319342 | 0.0033513 | 2 x 10^-21^ |
| 31 | rs2287623 | G | A | 0.0252441 | 0.00343616 | 2 x 10^-13^ |
| 32 | rs2569550 | T | C | -0.0489645 | 0.00352037 | 6 x 10^-44^ |
| 33 | rs2618566 | G | T | 0.0279288 | 0.00350837 | 2 x 10^-15^ |
| 34 | rs2638281 | A | G | -0.0277244 | 0.00354737 | 6 x 10^-15^ |
| 35 | rs2642438 | A | G | -0.0253638 | 0.00367292 | 5 x 10^-12^ |
| 36 | rs2721961 | T | G | 0.0319608 | 0.00369942 | 6 x 10^-18^ |
| 37 | rs2740488 | A | C | 0.0244647 | 0.00378703 | 1 x 10^-10^ |
| 38 | rs2792751 | T | C | 0.023245 | 0.00372325 | 4 x 10^-10^ |
| 39 | rs28601761 | C | G | 0.0651902 | 0.00340739 | 1 x 10^-81^ |
| 40 | rs28615248 | T | C | -0.0230417 | 0.00419995 | 4 x 10^-08^ |
| 41 | rs326 | A | G | 0.0245151 | 0.00366892 | 2 x 10^-11^ |
| 42 | rs34707604 | T | C | -0.0369893 | 0.00399395 | 2 x 10^-20^ |
| 43 | rs35081008 | C | T | 0.0316051 | 0.0049187 | 1 x 10^-10^ |
| 44 | rs35199321 | T | C | 0.0250488 | 0.00337964 | 1 x 10^-13^ |
| 45 | rs3756772 | C | T | -0.0226923 | 0.00341035 | 3 x 10^-11^ |
| 46 | rs3794695 | C | T | -0.0506558 | 0.00425573 | 1 x 10^-32^ |
| 47 | rs3808348 | C | T | 0.0286039 | 0.00409402 | 3 x 10^-12^ |
| 48 | rs3865314 | A | C | 0.0336493 | 0.00335015 | 1 x 10^-23^ |
| 49 | rs41279633 | G | T | -0.0462563 | 0.00459113 | 7 x 10^-24^ |
| 50 | rs4299376 | G | T | 0.0591949 | 0.00358045 | 2 x 10^-61^ |
| 51 | rs4307732 | G | A | -0.0540467 | 0.00543729 | 3 x 10^-23^ |
| 52 | rs4689640 | G | A | -0.0222329 | 0.00359965 | 7 x 10^-10^ |
| 53 | rs472495 | G | T | -0.047615 | 0.00352254 | 1 x 10^-41^ |
| 54 | rs4738684 | A | G | 0.0264075 | 0.0035303 | 7 x 10^-14^ |
| 55 | rs4757676 | C | T | -0.0211313 | 0.00378931 | 3 x 10^-08^ |
| 56 | rs4757676 | C | T | -0.0211313 | 0.00378931 | 3 x 10^-08^ |
| 57 | rs4841132 | A | G | -0.0532143 | 0.00576841 | 3 x 10^-20^ |
| 58 | rs557933 | A | C | -0.0373711 | 0.00336961 | 1 x 10^-28^ |
| 59 | rs55843714 | C | T | -0.0187464 | 0.00338513 | 3 x 10-^08^ |
| 60 | rs55938402 | G | A | 0.0439218 | 0.00558999 | 4 x 10^-15^ |
| 61 | rs562338 | A | G | -0.108021 | 0.00437318 | 1 x 10^-134^ |
| 62 | rs564449 | T | G | 0.0321075 | 0.00515145 | 5 x 10^-10^ |
| 63 | rs58542926 | C | T | 0.113287 | 0.00657525 | 2 x 10^-66^ |
| 64 | rs6093446 | G | A | -0.0242379 | 0.00368753 | 5 x 10^-11^ |
| 65 | rs62117160 | G | A | 0.313627 | 0.00816507 | 1 x 10^-200^ |
| 66 | rs633695 | A | G | -0.0261983 | 0.00367788 | 1 x 10^-12^ |
| 67 | rs6602911 | C | T | -0.0195706 | 0.00347388 | 2 x 10^-08^ |
| 68 | rs6709904 | A | G | 0.0377578 | 0.00530905 | 1 x 10^-12^ |
| 69 | rs6882076 | T | C | -0.0352043 | 0.00345053 | 2 x 10^-24^ |
| 70 | rs695983 | G | A | -0.0227399 | 0.00367704 | 6 x 10^-10^ |
| 71 | rs7138498 | T | C | -0.023091 | 0.00409674 | 2 x 10^-08^ |
| 72 | rs71490145 | C | T | 0.0322136 | 0.00580191 | 3 x 10^-08^ |
| 73 | rs7202323 | T | G | 0.0234485 | 0.00396882 | 4 x 10^-09^ |
| 74 | rs7254892 | G | A | 0.402765 | 0.00998435 | 1 x 10^-200^ |
| 75 | rs72631343 | C | G | 0.0320859 | 0.00499254 | 1 x 10^-10^ |
| 76 | rs72694393 | G | T | -0.0207422 | 0.00335488 | 6 x 10^-10^ |
| 77 | rs72805692 | A | G | 0.0285443 | 0.00520986 | 4 x 10^-08^ |
| 78 | rs73066485 | T | G | -0.0332788 | 0.00401444 | 1 x 10^-16^ |
| 79 | rs73620883 | G | A | 0.0292586 | 0.00505388 | 7 x 10^-09^ |
| 80 | rs7528419 | A | G | 0.126817 | 0.00404211 | 1 x 10^-200^ |
| 81 | rs7534572 | C | G | -0.0426769 | 0.00350281 | 4 x 10^-34^ |
| 82 | rs7538475 | C | T | 0.0260022 | 0.00472724 | 4 x 10^-08^ |
| 83 | rs7620985 | G | A | -0.033947 | 0.00560441 | 1 x 10^-09^ |
| 84 | rs76895963 | T | G | 0.0744413 | 0.0128872 | 8 x 10^-09^ |
| 85 | rs77542162 | A | G | -0.151187 | 0.0111453 | 7 x 10^-42^ |
| 86 | rs780093 | T | C | 0.0387599 | 0.00345828 | 4 x 10^-29^ |
| 87 | rs78784525 | G | T | 0.0257183 | 0.00363211 | 1 x 10^-12^ |
| 88 | rs79220007 | T | C | 0.0426947 | 0.00620443 | 6 x 10^-12^ |
| 89 | rs79600321 | C | T | -0.0548679 | 0.00998193 | 4 x 10^-08^ |
| 90 | rs8019852 | G | A | -0.021664 | 0.00335052 | 1 x 10^-10^ |
| 91 | rs8069105 | T | G | 0.0226479 | 0.00355683 | 2 x 10^-10^ |
| 92 | rs821840 | A | G | 0.0353255 | 0.00355282 | 3 x 10^-23^ |
| 93 | rs9306897 | T | C | 0.0392515 | 0.00352011 | 7 x 10^-29^ |
| 94 | rs9402685 | T | C | 0.0249326 | 0.0037986 | 5 x 10^-11^ |
| 95 | rs964184 | G | C | 0.0784353 | 0.0049187 | 3 x 10^-57^ |
| 96 | rs9686661 | C | T | -0.0233814 | 0.00419706 | 3 x 10^-08^ |
| 97 | rs9875338 | G | A | 0.0222434 | 0.00338747 | 5 x 10^-11^ |

Information on genetic variants used for polygenic risk score (UK Biobank 2) in the UK Biobank used in Supplementary Figure 4. SE = Standard error.

## Supplementary Figure 5.


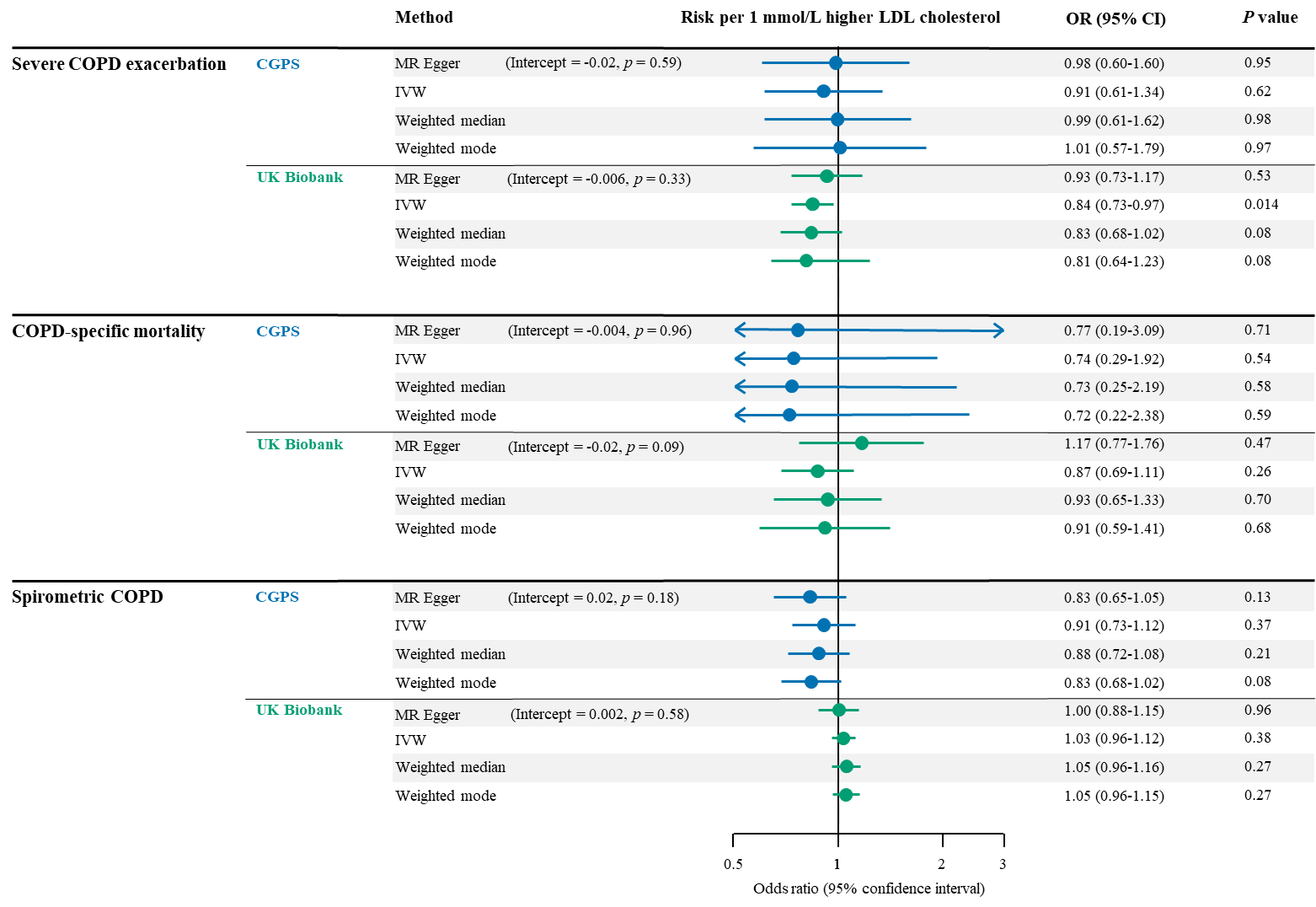


Causal risk ratios using MR Egger, inverse-variance weighted (IVW), weighted median, and weighted mode methods of COPD outcomes per 1 mmol/L (39 mg/dL) higher genetically LDL cholesterol in the Copenhagen General Population Study (CGPS) and UK Biobank. Analyses in the UK Biobank were adjusted for 10 principal components and genotype chip.

## Supplementary Figure 6.


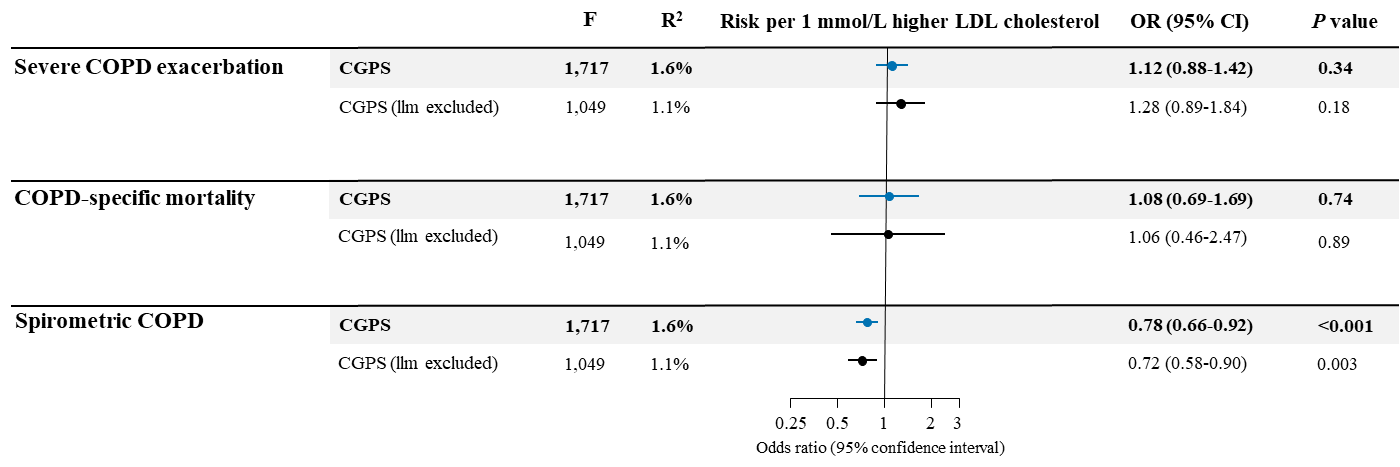


Risk of COPD outcomes per 1 mmol/L (39 mg/dL) higher genetically determined LDL cholesterol with and without individuals receiving lipid-lowering medication (llm) excluded in the Copenhagen General Population Study (CGPS). Odds ratios for genetically high LDL cholesterol were calculated by instrumental variable analysis. Highlighted estimates indicate the primary analyses used in the main manuscript. F: the strength of the genetic instrument; R^2^: percent contribution of genetic instrument to the variation in LDL cholesterol.
